# Supplementary material for: Impact of Invasive Fungal Diseases on Survival under Veno-Venous Extracorporeal Membrane Oxygenation for ARDS
Source: J Clin Med. 2022 Mar 31;11(7):1940. doi: 10.3390/jcm11071940 (PMC8999842; doi:10.3390/jcm11071940)
Supplement: Supplementary file 1 [file jcm-11-01940-s001.zip › Table S1.pdf]

Invasive Fungal Disease:	Yes<br>N (%)	No<br>N (%)
Charlson Comorbidity Index Items		
- Myocardial Infarction	1 (4.5)	2 (9.1)
- Congestive Heart Failure	2 (9.1)	1 (4.5)
- Peripheral Vascular Disease	2 (9.1)	1 (4.5)
- Cerebrovascular Disease	3 (13.6)	1 (4.5)
- Dementia	0 (0)	0 (0)
- Chronic Pulmonary Disease	4 (18.2)	5 (22.7)
- Connective Tissue Disease	0 (0)	0 (0)
- Peptic Ulcer Disease	0 (0)	1 (4.5)
- Mild Liver Disease	1 (4.5)	1 (4.5)
- Diabetes (without end organ damage)	4 (18.2)	2 (9.1)
- Hemiplegia	0 (0)	0 (0)
- Moderate or Severe Renal Disease	1 (4.5)	0 (0)
- Diabetes (with end organ damage)	1 (4.5)	1 (4.5)
- Any Tumor	2 (9.1)	2 (9.1)
- Leukemia	0 (0)	0 (0)
- Lymphoma	0 (0)	0 (0)
- Moderate of Severe Liver Disease	0 (0)	0 (0)
- Metastatic Solid Tumor	0 (0)	0 (0)
- AIDS	0 (0)	0 (0)
Surgery *	4 (18.2)	5 (22.7)
CRRT *	14 (63.6)	15 (68.2)
Immunosuppression **	4 (18.2)	6 (27.3)

Table S1. Comorbidities and potentially predisposing factors for invasive fungal disease (IFD) in patient cohorts. CRRT: Continuous Renal Replacement Therapy. * During current hospitalization, before or at time of IFD diagnosis. ** As defined in text. There were no statistically significant differences between patients with IFD and without IFD.
