# Supplementary material for: Impact of Invasive Fungal Diseases on Survival under Veno-Venous Extracorporeal Membrane Oxygenation for ARDS
Source: J Clin Med. 2022 Mar 31;11(7):1940. doi: 10.3390/jcm11071940 (PMC8999842; doi:10.3390/jcm11071940)
Supplement: Supplementary file 1 [file jcm-11-01940-s001.zip › Table S2.pdf]

Pathogen	Location	1st treatment	2nd treatment	Sensible to treatment	Time (d) between						Death	Treatment at time of death/discharge
					ECMO - IFD	ECMO - IFDval	IFD - treatment	IFDval - treatment	clearance - end of antifungals	IFD - clearance		
C. albicans	Blood	Voriconazole		Yes	0	6	1	-5	17	7	Yes	No
C. albicans	Blood	Fluconazole	Caspofungin	Yes	18	25	1	-6	n/a	4	Yes	Yes
C. glabrata	Blood	Fluconazole	Anidulafungin	unknown	12	16	-10	-14	n/a	never	Yes	Yes
C. albicans	Blood	Voriconazole	Anidulafungin, Fluconazole	Yes	6	11	0	-5	n/a	1	No	Yes
C. krusei	Blood	Caspofungin		Yes	14	17	0	-3	n/a	never	Yes	Yes
C. albicans	Blood	Caspofungin		Yes	29	34	2	-3	17	5	Yes#	No
C. albicans	Blood	Caspofungin	Fluconazole	Yes	0	5	2	-3	15	3	Yes#	Yes
C. albicans	Blood	Caspofungin	Fluconazole	Yes	0	7	2	-5	14	5	No	No
C. albicans	Blood	Caspofungin	Anidulafungin	Yes	23	27	-1	-5	n/a	2	Yes	Yes
C. albicans	Blood	Caspofungin	Fluconazole	Yes	0	4	1	-3	13	4	No	No
C. albicans	Blood	Voriconazole	Caspofungin	Yes	0	4	2	-2	14	4	No	No
C. albicans	Blood	Caspofungin	Fluconazole	Yes	14	18	2	-2	n/a	3	Yes	Yes
C. albicans	Blood	n/a *		n/a *	11	16	n/a *	n/a *	n/a *	never	Yes *	n/a *
C. albicans	Blood	Caspofungin		Yes	8	14	2	-4	n/a	never	Yes	Yes
C. albicans	Blood	Voriconazole	Fluconazole	Yes	41	45	2	-2	n/a	never	Yes #	Yes
C. albicans	Blood	Voriconazole	Anidulafungin	Yes	0	6	0	-6	18	2	Yes	No
C. albicans	Blood	Caspofungin		Yes	23	29	2	-4	n/a	never	Yes	Yes
C. kefyr	Blood	Caspofungin	Voriconazole	Yes	18	22	1	-3	n/a	never	Yes	Yes
Asp. terreus	BALF	Voriconazole		Yes	3	10	3	-4	n/a	never	Yes	Yes
C. krusei	Blood	Amphotericin B (lipo.)		Yes	13	16	0	-3	n/a	never	Yes	Yes
C. parapsilosis	Blood	Caspofungin		Yes	36	42	2	-4	n/a	1	Yes	Yes
Asp. fumigatus	BALF	None **		Yes	12	22	n/a	n/a	n/a	never	Yes	No

Table S2. Diagnosis of invasive fungal disease (IFD) and treatment in patients on ECMO support. Asp.: Aspergillus. BALF: Bronchoalveolar lavage fluid. C.: Candida. IFDval.: Validated fungal culture and resistogram. n/a: not applicable. Lipo.: Liposomal. * death occurred at time of IFI diagnosis. ** Test results became available after patient had deceased. # Weaning of ECMO completed before death
